# Extraction, HPTLC Analysis and Antiobesity Activity of *Jatropha tanjorensis* and *Fraxinus micrantha* on High-Fat Diet Model in Rats

**DOI:** 10.3390/life13061248

**Published:** 2023-05-25

**Authors:** Swati Srivastava, Tarun Virmani, Md. Rafiul Haque, Abdulsalam Alhalmi, Omkulthom Al Kamaly, Samar Zuhair Alshawwa, Fahd A. Nasr

**Affiliations:** 1 School of Pharmaceutical Sciences, MVN University, Palwal 121105, India; swatisrivastva186@gmail.com; 2 School of Pharmacy, Al-Karim University, Katihar 854106, India; hrafiul@gmail.com; 3 Department of Pharmaceutical Sciences, College of Pharmacy, Aden University, Aden 6312, Yemen; 4 Department of Pharmaceutical Sciences, College of Pharmacy, Princess Nourah bint Abdulrahman University, P.O. Box 84428, Riyadh 11671, Saudi Arabia; omalkmali@pnu.edu.sa (O.A.K.); szalshawwa@pnu.edu.sa (S.Z.A.); 5 Department of Pharmacognosy, College of Pharmacy, King Saud University, Riyadh 11451, Saudi Arabia; fnasr@ksu.edu.sa

**Keywords:** *Jatropha tanjorensis*, *Fraxinus micrantha*, HPTLC, anti-obesity activity, high-fat diet model, histological

## Abstract

The accumulation of body fat due to an imbalance between calorie intake and energy expenditure is called obesity. Metabolic syndrome increases the risk of heart disease, type 2 diabetes, and stroke. The purpose of this study was to determine the effect of *Jatropha tanjorensis* (J.T.) and *Fraxinus micrantha* (F.M.) leaf extracts on high-fat diet-induced obesity in rats. Normal control, high-fat diet (HFD) control, orlistat standard, and test groups were created using male Albino Wistar rats (n = 6 per group) weighing 190 ± 15 g. Except for the control group, all regimens were administered orally and continued for 6 weeks while on HFD. Evaluation criteria included body weight, food intake, blood glucose, lipid profile, oxidative stress, and liver histology. High-Performance Thin Layer Chromatography (HPTLC) analysis was performed using a solvent system (7:3 hexane: ethyl acetate for sitosterol solution and *Jatropha tanjorensis* extracts and 6:4 hexane: ethyl acetate: 1 drop of acetic acid for esculetin and *Fraxinus micrantha* extracts). There were no deaths during the 14 days before the acute toxicity test, indicating that aqueous and ethanolic extracts of both J.T. and F.M. did not produce acute toxicity at any dose (5, 50, 300, and 2000 mg/kg). The ethanolic and aqueous extracts of J.T. and F.M. leaves at 200 and 400 mg/kg/orally showed a reduction in weight gain, feed intake, and significant decreases in serum glucose and lipid profile. As compared to inducer HFD animals, co-treatment of aqueous and ethanolic extract of both J.T. and F.M. and orlistat increased the levels of antioxidant enzymes and decreased lipid peroxidation. The liver’s histological findings showed that the sample had some degree of protection. These results indicate that ethanolic samples of J.T. have antidiabetic potential in diabetic rats fed an HFD. The strong antioxidant potential and restoration of serum lipid levels may be related to this. Co-treatment of samples JTE, JTAQ, FME, FMAQ and orlistat resulted in an increase in antioxidant enzymes and reduction in lipid peroxidation as compared to inducer HFD animals. We report, for the first time, on using these leaves to combat obesity.

## 1. Introduction

With more than 312 million clinically obese people worldwide, obesity is one of the most serious public health problems of the 21st century [1]. One of the hallmarks of obesity is the accumulation of fat in adipose tissue and other internal organs [2,3,4]. Many chronic diseases can be caused by obesity, including type 2 diabetes, heart disease, hyperlipidemia, atherosclerosis, certain cancers, and osteoarthritis [5]. Obesity, along with high social and health costs, increases morbidity and mortality worldwide. As a result, several attempts are being made to find anti-obesity drugs worldwide [6,7,8].

Consumers are drawn to naturally derived anti-obesity products due to the widespread belief that anything natural should be more effective and safer than traditional treatments. Sibutramine, orlistat, and rimonabant in particular have been associated with numerous serious side effects, including gastrointestinal problems and serious cardiovascular side effects [9]. Therefore, the current search for new anti-obesity drugs derived from natural sources due to their favorable pharmacological profile and low side effects has become the current strategy [10]. In wet forest areas of West Africa, *Jatropha tanjorensis* J. L. Ellis & Saroja (Euphorbiaceae) are typical weeds found in crops, shrub shoots, roadsides, and disturbed areas [11]. As a hybrid, it is a perennial herb with an intermediate phenotype between *Jatropha curcas* and *Jatropha gossypifolia* [12,13]. Catholic Vegetable, Jatropha, Hospital Too Far, and Jana Ifaya are just some of the common names [14,15]. In veterinary medicine, as well as in traditional and folk medicine, all parts of the plant, including the seeds, leaves, and bark, are used regardless of whether they are used fresh or in decoction form. According to early research, the antioxidant minerals phosphorus, selenium, zinc, and vitamins C and E are found in abundance in the *Jatropha tanjorensis* plant [16]. Phytochemical analysis of *Jatropha tanjorensis* leaves has revealed the presence of biologically active constituents such as alkaloids, flavonoids, tannins, cardiac glycosides, anthraquinones, and saponins [17,18]. The leaves of the plant have been widely used in tonics and soups originally from Nigeria and are known to increase blood volume. An infusion of *Jatropha tanjorensis* leaves is taken orally in southwestern Nigeria to treat symptoms of diabetes. The antidiabetic activities of the J.T. ethanolic extracts (JTE), namely JTE chloroform, JTE ethylacetate, JTE aqueous, have been evaluated [19]. The antibacterial and anti-inflammatory effects of leaf extracts in hexane, chloroform, and methanol are different [20].

The leaves and stems of *Jatropha curcas* and *Jatropha tanjorensis* have been studied from morphological and anatomical points of view [18,21]. Acute and subacute toxicity and microscopic examination of *Jatropha tanjorensis* leaves have been described [22], as well as the antibacterial activity of aqueous leaf extracts [23]. A versatile temperate deciduous tree species of the Himalayas of outstanding medicinal potential and ethnobotanical importance is *Fraxinus micrantha*, Lingelsh (also known by its native name Angu, English name Ash and surname Oleaceae). *Fraxinus micrantha* is one of the ashes found in Asia, mainly in India and Nepal. It can be found in the Indian states of Himachal Pradesh and Uttar Pradesh [24].

Since ancient times, *Fraxinus micrantha* has been researched for both its medicinal and economic benefits. The inner bark infusion is used locally by Dharchula, Himalayan residents to cure liver enlargement, jaundice, and other liver ailments [25]. Due to the presence of numerous glycosides, including fraxin, and an active diuretic agent called coumarin glycoside, Fraxinus species have been employed in folk medicine for their purgative and diuretic effects. Additionally, the leaves and the bark are used to cure cystitis, rheumatoid arthritis, constipation, and itchy scalps [26]. The discovery of the secoiridoid glucosides, which are significant metabolites in the genus of the family Oleaceae, has led to an increase in interest in the phytochemistry of Fraxinus in recent years [27].

Because of their similarity to human obesity and associated metabolic effects, the animal model of diet-induced obesity is one of the most widely used and reliable models for obesity research. The result is increased food intake, weight gain, body fat accumulation, impaired lipid profile, lack of antioxidant stability, and increased insulin resistance parameters [28,29]. Therefore, this study aimed to evaluate the effects of alcoholic and aqueous extracts of *Jatropha tanjorensis* and *Fraxinus micrantha* leaves on high-fat diet-induced obesity in rats.

## 2. Materials and Methods

### 2.1. Plant Material 

Fresh leaves of *Jatropha tanjorensis* were collected from near Coimbatore, India, while the leaves of *Fraxinus micrantha* were collected in May from Nainital, Kumaun, Himalayas, India. Both drugs were identified from Vital Herbs, Uttam Nagar, Delhi. The leaves were dried at a constant weight in the air at room temperature, and then the leaves were crushed.

### 2.2. Chemical Reagents

All chemicals used in this study were obtained from the Hi Media Laboratories Pvt. Ltd. (Mumbai, India), Sigma-Aldrich Chemical Co. (Milwaukee, WI, USA), SD Fine-Chem. Ltd. (Mumbai, India), and SRL Pvt. Ltd. Chlorpheniramine maleate and clonidine were obtained from Unichem, Ltd. (Alchem, Mumbai). Only analytical grade compounds were used in the study.

### 2.3. Extract

#### 2.3.1. Ethanol Extract

A total of 150 gm of powdered leaves of F.M. and 50 gm of dry powder leaves of J.T. were placed in a Soxhlet device with a thimble. An organic solvent was used for extraction, i.e., ethanol, for 8–10 h and the temperature of the mantle heater was adjusted to 40–60 °C. After the extraction process, the sample extract was filtered and concentrated to dryness. Extracts were collected in sealed containers [30]. Yields of all extracts were calculated.

#### 2.3.2. Water Extract

Coarse powder of leaves (150 g of J.T. and 50 g of F.M.) was boiled in distilled water for 15 min. After leaving this at room temperature for 15 min, it was filtered through a muslin cloth. The resulting solution was boiled again to obtain a thick concentrated extract. After drying, the extract was collected in a sealed container [31]. Extraction yields of all extracts were calculated.

### 2.4. High-Performance Thin Layer Chromatography (HPTLC) 

HPTLC was performed on silica gel 60 F_254_ 100 × 100 mm plates (Merck) with hexane: ethyl acetate (7:3 *v*/*v*) as mobile phase for the standard (β-sitosterol) solution (2–10 µL) and J.T. (0.2–0.3 µL); and hexane: ethyl acetate: 1 drop of acetic acid (6:4 *v*/*v*) as mobile phase for the standard (esculetin) solution (1–4 µL) and F.M. (1–3 µL). These were applied to the plate as 8 mm bands. Application of the sample was performed with CAMAG-Linomat 5 Automated spray on a band applicator equipped with a 100 µL syringe and operated with the settings: band length 8 mm, application rate 150 nL/s, application volume 0.20 µL, distance between track 14.4 mm, distance from the plate side edge 15.0 mm and solvent front position 70 mm. CAMAG TLC visualizes 2 and was used densitometrically to scan the bands. The scanner operating parameters were set to mode absorption/reflection at an optimized wavelength of 254, 366 nm, and in the visible range. Integration parameters were set to gauss (legacy) with sensitivity 0.1, separation 1, and threshold 0.1. 

### 2.5. HFD-Induced Obesity

Albino Wistar rats weighing 190 ± 15 g obtained from the breeding farm of the Pinnacle Biomedical Research Institute (PBRI), Bhopal, were selected. They were then divided into groups of 6 with controlled temperature and humidity (25 ± 2 °C, 55–65%). Rats were provided regular rodent chow and unlimited water. Adult male Wistar rats were acclimated to laboratory conditions for 2 weeks.

All studies were conducted indoors without background noise. Each study set used a different group of rats (n = 6). The Institutional Animal Ethics Committee (IAEC) of the Pinnacle Biomedical Research Institute (PBRI) approved animal studies by Bhopal, India (Reg. No. 1824/PO/ERe/S/15/CPCSEA). The protocol approval reference number is PBRI/IAEC/10-09-22/012. A normal control group (NC) of 6 rats was randomly divided and fed a typical diet. Afterwards, animals in different experimental groups were given sufficient food and water along with a high-fat diet for 6 weeks. The high-fat content was obtained by mixing coconut oil with vanaspati Indian ghee in a ratio of 3:1 (*v*/*v*). Rats were fed daily at a dose of 3 mL per kg of body weight.

### 2.6. Acute Toxicity Studies

J.T. and F.M.’s acute toxicity investigation was completed according to the Organisation for Economic Cooperation and Development (OECD) guidelines [32]. Four treatment groups with dosages of 5 mg/kg, 50 mg/kg, 300 mg/kg, and 2000 mg/kg body weight were included in the test groups. Using an appropriate intubation canula or a specifically developed oral needle, the test drug was gavaged in a single dose. Before dosing, animals were fasted for three hours (only food was withheld for 3 h but not water). The animals were closely monitored for behavioral changes, mortality, and appearance starting in the first four hours, then occasionally over the next twenty-four hours, and finally daily for a period of two weeks up to fourteen days [33].

### 2.7. Experimental Design

Wistar rats were divided into control and preventive groups with at least six animals each.

Group I: normal control; rats were administered saline/vehicle.

Group II: HFD Control; animals were fed a standard granulated diet with an HFD of 3 mL daily for 6 weeks.

Group III: Standard Medication Treatment; animals were fed a standard granular diet with 3 mL of HFD daily for 6 weeks. After 3 weeks of the study, 30 mg/kg/day of orlistat was administered after 3 weeks of HFD, and this continued for the remaining 3 weeks; this was regarded as the standard group.

Group IV: Animals were fed a standard granular diet with an HFD of 3 mL daily for 6 weeks. After 3 weeks of the study, HFD was fed for 3 weeks, then JTAQ 200 mg/kg/day was administered and continued until the remaining 3 weeks; this was considered a treatment group.

Group V: Animals were fed a standard granulated diet with an HFD of 3 mL daily for 6 weeks. After 3 weeks of the study, HFD was fed for 3 weeks, then JTAQ 400 mg/kg/day was administered and continued until the remaining 3 weeks; this was considered a treatment group.

Group VI: Animals were fed a standard granular diet with 3 mL of HFD daily for 6 weeks. After 3 weeks of the study, 200 mg/kg/day of JTE was administered after 3 weeks of HFD and continued for the remaining 3 weeks; this was considered a treatment group.

Group VII: Animals were fed a standard granulated diet with an HFD of 3 mL daily for 6 weeks. After 3 weeks of study, 400 mg/kg/day of JTE was administered after 3 weeks of HFD and continued for the remaining 3 weeks; this was considered a treatment group.

Group VIII: Animals were fed a standard granulated diet with an HFD of 3 mL daily for 6 weeks.

After 3 weeks of the study, 200 mg/kg/day of FMAQ was administered after 3 weeks of high-fat diet food, and continued until the remaining 3 weeks; this was considered a treatment group.

Group IX: Animals were fed a standard granulated diet with an HFD of 3 mL daily for 6 weeks. After 3 weeks of the study, HFD was fed for 3 weeks, and FMAQ 400 mg/kg/day was administered, and continued until the remaining 3 weeks; this was considered a treatment group.

Group X: Animals were fed a standard granulated diet with an HFD of 3 mL daily for 6 weeks. After 3 weeks of the study, HFD was fed for 3 weeks, and FME 200 mg/kg/day was administered, and continued until the remaining 3 weeks; this was considered a treatment group.

Group XI: Animals were fed a standard granular diet containing 3 mL of an HFD for 6 weeks. After 3 weeks of the study, HFD was fed for 3 weeks, then PME 400 mg/kg/day was administered and continued until the remaining 3 weeks; this was considered a treatment group.

### 2.8. Study Parameters

Body weight, blood glucose, lipid profile, oxidative stress, and liver histology were investigated as variables. After animals were sacrificed at the end of the study, blood parameters and oxidative stress markers were examined. Then, the liver was washed thoroughly with ice-cold saline and Tris-HCl buffer (0.1 M, pH 7.4) for antioxidant assay. Total cholesterol (TC) and triglyceride (TG) levels were assessed using a commercial assay kit (SPAN Diagnostics Ltd., Surat, India) according to the manufacturer’s instructions. High-density lipoprotein (HDL) levels were measured using the HDL test kit (Reckon Diagnostics Pvt. Ltd.,. Baroda, India). The Friedewald equation was used to calculate the concentration of LDL [34].

#### 2.8.1. LDL Level

Serum LDL level (mg/dl) = total cholesterol − (HDL level + VLDL level)

#### 2.8.2. Oxidation Markers

##### Superoxide Dismutase (SOD)

The red formazan dye reduction process produces superoxide radicals, which are detected by the superoxide dismutase assay kit using a tetrazolium salt. One unit (U) of SOD activity is the amount of enzyme required to demonstrate 50% superoxide radical dismutation. In a nutshell, 0.1 mL of sample was introduced together with 1.2 mL of buffer containing 0.052 M sodium pyrophosphate, 186 µM phenazine methosulphate, 300 µM nitroblutetrazolium, and 0.2 µM NADH, with 90 s of incubation time at 30 °C. Glacial acetic acid, 0.1 mL, was added and then we added 4.0 mL of n-butanol and stirred. After 10 min of standing time, the butanol layer and centrifuge were separated. At 560 nm, the reduction was measured, and the percentage of SOD inhibition in comparison to the control was calculated. Unit U/mg tissue was used to determine and express one unit of SOD [35].

##### Malondialdehyde (MDA)

Through the measurement of thiobarbituric acid reactive material, liver peroxidation was discovered (TBARS). In a nutshell, 0.2 mL of an aliquot had 0.2 mL 8.1% SDS, 1.5 mL 20% acetic acid, and 1.5 mL 8% TBA added to it (made up to a volume of 4 mL with distilled water). Using a glass ball as the condenser, this was heated in a water bath for 60 min, then cooled and made up to a volume of 5 mL. Then, 5 mL of butanol: pyridine was added (15:1). The mixture was centrifuged at 3000 rpm for 10 min after being vortexed for 2 min. Then we removed the top layer of the mixture, and then used a UV-Vis Spectrophotometer to measure the pink product’s absorbance at 532 and 600 nm wavelengths (Systronic-2202). The difference in absorbance was measured and contrasted with that of standard solutions of varying concentrations of malonaldehyde tetramethyl acetal. Nmol MDAm/g tissue was used to express MDA activity [36].

##### Reduced Glutathione (GSH)

Through a linked reaction with GR, the glutathione peroxidase test kit indirectly assesses GPx activity. Glutathione becomes oxidized when hydroperoxide is reduced by GPx; this oxidized glutathione is then recycled to its reduced state by GR and NADPH. The absorbance falls as NADPH is converted to NADP+ through oxidation. A total of 10% by weight of tissue homogenate in a pH 7.4 phosphate buffer solution, 1.5 mL of 20% TCA and 1.5 mL of 1 mM EDTA were combined with 0.2 mL of homogenate and left to sit for 15 min. Then it received 10 min of centrifuging at 2000 rpm. The supernatant (400 L) was collected, and the supernatant was transferred to a fresh tube containing 1.8 mL of Elman’s reagent (0.1 mM 5,5′-dithiol bis-2-nitrobenzoic acid produced in 0.3 M phosphate buffer, pH 7 with 1% sodium citrate solution; keep at 0–4 °C in the dark). Distilled water was used to maintain volumes of up to 2 mL. At 412 nm, the absorbance was measured [37].

#### 2.8.3. Histopathology

Liver samples were immersed in a 10% formalin solution for histological analysis. These tissues were prepared, dehydrated with different concentrations of alcohol, washed with toluene, and immersed in molten paraffin wax for specified times. Freshly melted paraffin wax was used to pour the treated fabric and then the wax was allowed to harden. To demonstrate the general structure of the tissue, sections were produced at 3 μm thickness, dried on a hot plate for 15 min, and then stained with hematoxylin and 1% eosin aqueous solution. Stained glass slides were dehydrated in alcohol of varying strength, then purified in xylene and embedded in Canadian balsam. Sections were examined up close using a ×10 objective.

### 2.9. Statistical Analysis

Data are presented as mean standard deviation (n = 6). Results were statistically tested using a one-way analysis of variance (ANOVA) followed by Bonferroni’s *t*-test. When comparing the two groups, the significance level was *p* < 0.05.

## 3. Results

**Figure 1:**
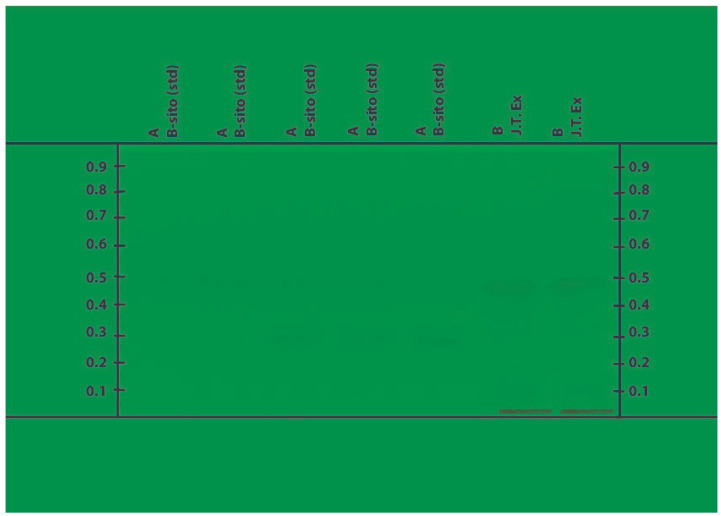
Chromatogram obtained from separation of J.T extract and visualized under UV light of wavelength 254 nm.

**Figure 2:**
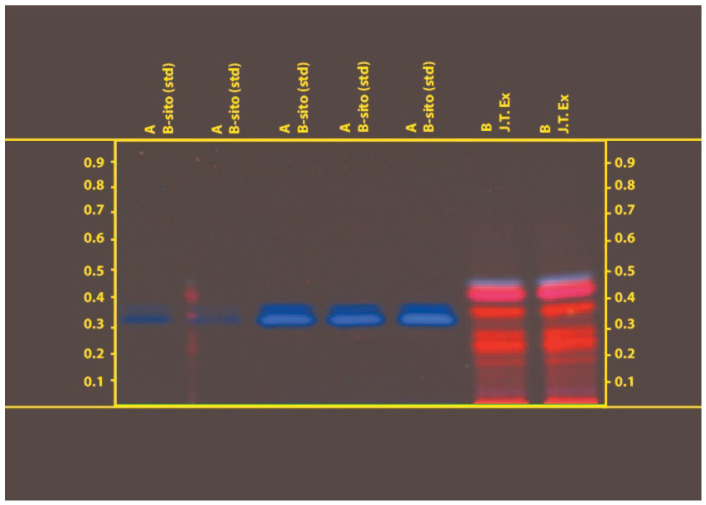
Chromatogram obtained from the separation of J.T extract and visualized under UV light of wavelength 366 nm.

**Figure 3:**
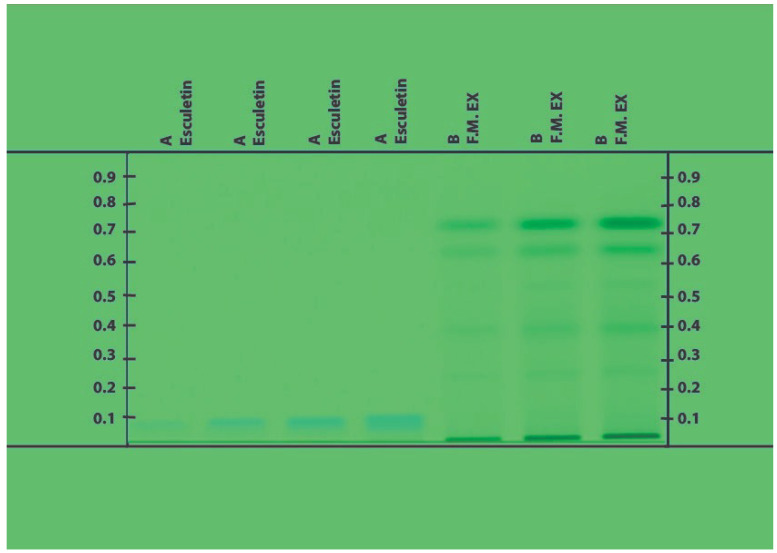
Chromatogram obtained from the separation of F.M extract and visualized under UV light of wavelength 254 nm.

**Figure 4:**
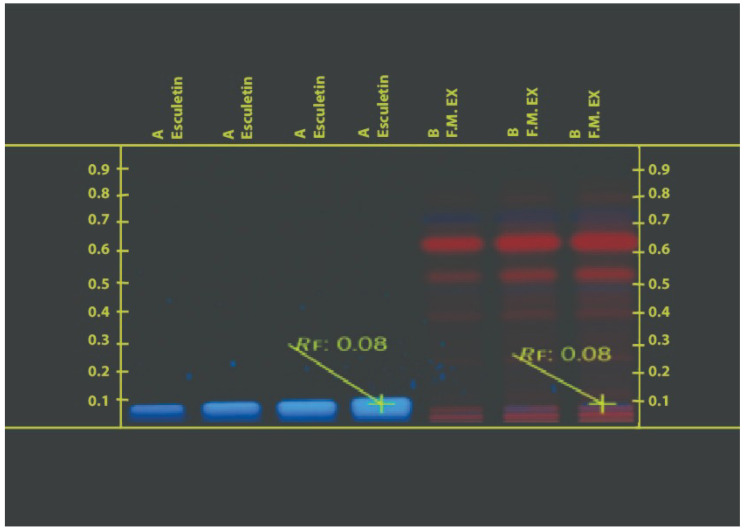
Chromatogram obtained from the separation of F.M extract and visualized under UV light of wavelength 366 nm.

To achieve the real yield of extraction, the crude extracts produced after each successive Soxhlet extraction and decoction technique were concentrated in a water bath by entirely evaporating the solvents. The percentage yields of the resultant extracts were determined to be 2.20 and 0.83% for J.T. and 10.0 and 12.6% for F.M. in various solvents such as ethanol and water. At 254 and 366 nm, the J.T. ethanolic extract HPTLC chromatogram data were examined. Six spots were seen in the extract, and their Rf values were 0.45, 0.38, 0.28, 0.22, 0.19, and 0.01. Figure 1 and Figure 2 show that the standard (-sitosterol) Rf value was 0.33. Analysis of the F.M. extract HPTLC chromatogram results was carried out at 254 and 366 nm. The chromatogram showed nine spots for the extract at 0.8, 0.76, 0.65, 0.55, 0.40, 0.24, 0.08, 0.04, and 0.06 and one spot for the standard at 0.08 (esculetin). The outcomes demonstrated that esculetin was present in the F.M. extract (Figure 3 and Figure 4). No deaths occurred throughout the 2 weeks of the acute toxicity research, proving that the administration of Sample-JT and FM did not, at any dose, produce toxicity (5, 50, 300 and 2000 mg/kg). None of the treatment groups experienced any discernible weight loss throughout the 14-day observation period. The rats showed no indication of any abnormalities. Test samples JT and FM did not significantly alter the parameters tested (5, 50, 300, and 2000) such as urine, convulsions, tremors, changes in skin color, etc. 

Therefore, based on the most recent findings from an acute toxicity study, the final doses chosen for further research were 1/10th and 1/5th of 2000 mg/kg bw. At the beginning of the trial, the mean body weights of the seven experimental groups were comparable. The initial body weights (i.e., initial body weights) of the healthy control rats in Group I were normal, and they continued to be so for the next six weeks of the trial. After the trial, HFD-treated rats (Group II) showed a significantly higher body weight than the healthy control group (Group I) (*p* < 0.05). After receiving treatment with test samples JTAQ, JTE, FMAQ, and FME for six weeks, the changes in body weight to normal body weight were significantly (*p* < 0.05) maintained. However, compared to the HFD control group, therapy with the common medicine orlistat (30 mg/kg, p.o.) once daily for six weeks dramatically reduced body weight and feed intake. When compared to the HFD control group, once-daily treatment for six weeks with JTAQ, JTE, FMAQ, and FME significantly reduced body weight and feed intake (Table 1 and Table 2). 

**Figure 5:**
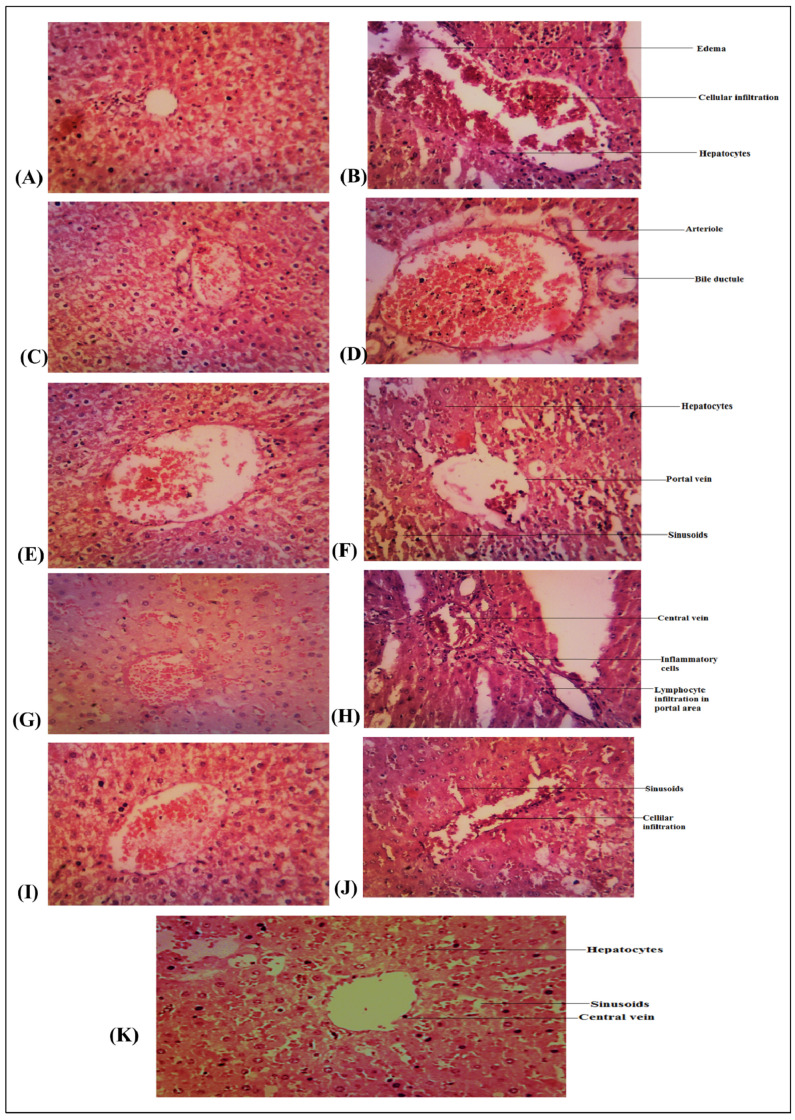
(**A**) Group I: Normal control (vehicle-treated), (**B**) Group II: HFD induced, (**C**) Group III: Standard orlistat treated, (**D**) Group IV: JTAQ 200 mg/kg, (**E**) Group V: JTAQ 400 mg/kg, (**F**) Group VI: JTE 200 mg/kg, (**G**) Group VII: JTE 400 mg/kg, (**H**) Group VIII: FMAQ 200 mg/kg, (**I**) Group IX: FMAQ 400 mg/kg, (**J**) Group X: FME 200 mg/kg, (**K**) Group XI: FME 400 mg/kg.

Throughout the trial, the serum glucose levels of the HFD control group significantly increased. The HFD control group (Group II) demonstrated increased blood glucose, indicative of impaired glucose tolerance, whereas HFD animals receiving treatment with orlistat, JTE, and FME (Group III) demonstrated decreased blood glucose levels in comparison to the HFD-treated group, except JTAQ, FMAQ treated animals, which displayed less effective outcomes (Table 3). When compared to normal control rats, the HFD inducer group (Group II) showed a two-fold rise in the level of TGs (Group I). When compared to the HFD inducer group (Group II), the serum TG concentrations of the rats in Group IV-XI treated for 6 weeks with JTAQ, JTE, FMAQ, and FME decreased, respectively (*p* < 0.05). As compared to the HFD inducer group (Group II), TC levels were also affected in Group IV-XI treated rats (JTAQ, JTE, FMAQ, and FME) and decreased dose-dependently (*p* < 0.05). Additionally, JTE and FME treatment for six weeks in HFD rats (Groups VII and XI) resulted in significant (*p* < 0.05) reductions in LDL (44.53 ± 6.396 and 58.06 ± 7.510, respectively), in comparison to the HFD inducer group (99.65 ± 3.879) (Group II). When compared to control rats, the HFD inducer group (Group II) showed a drop (15.69 ± 0.667) in HDL (Group I). Contrarily, administration of JTAQ, JTE, FMAQ, and FME at 200 and 400 mg/kg raised HDL levels in HFD rats (Table 4). As compared to the normal control group, there was a significant drop in reduced glutathione (GSH), superoxide dismutase (SOD), and an increase in malondialdehyde (MDA) in the HFD control group. When compared to the HFD control group, the once-daily oral dose of orlistat for six weeks combined with HFD significantly enhanced the levels of GSH and SOD with a decrease in MDA. Additionally, compared to the HFD control group and comparable to standard medication (orlistat) treatment, the once-daily treatment with JTAQ, JTE, FMAQ, and FME (200 and 400 mg/kg, p.o.) for six weeks dramatically reduced the levels of GSH and SOD with a drop in MDA. Distribution of the test sample JTAQ, JTE, FMAQ, and FME inactivity demonstrates that JTAQ, JTE, FMAQ, and FME at 200 and 400 mg/kg had free radical scavenging activity, which may operate favorably against pathological changes brought on by the presence of O2– and OH– (Table 5). Histology of the liver sections from normal control animals showed normal hepatic cells with well-preserved cytoplasm, a prominent nucleus and nucleolus, and a well-brought-out central vein. The liver sections of Group 2 animals showed hepatic cells with severe toxicity, degeneration, edema and necrosis of hepatocytes with hemorrhage (h) and destruction of hepatic sinusoids. Induced rats treated with JTAQ, JTE, FMAQ, and FME had livers that were intensely affected by fatty changes, sinusoidal feathery degeneration and necrosis, congestion in the central vein with fibrous tissue proliferation, ballooning, and severe hepatocyte degeneration, whereas rats treated with JTE had livers that more closely resembled normal hepatic structure (Figure 5). 

The present study reveals that co-treatment of samples JTE, JTAQ, FME, FMAQ and orlistat resulted in an increase in the antioxidant enzymes and reduction in lipid peroxidation as compared to inducer HFD animals. The fact that the extracts were seen to significantly lower serum total lipids, total cholesterol, and LDL cholesterol suggests that it can be used in hyperlipidemia. As compared to other extracts, the ethanolic extract of *Jatropha tanjorensis* (JTE) leaves exhibits a promising role in the control of high-fat-induced obesity and explains the traditional use of Jatropha tanjorensis leaves to treat cardiac diseases [38]. In future research, we are planning to conduct GC-MS analysis, isolation and purification of various active ingredients present in the plants responsible for various kinds of pharmacological activities.

## 4. Conclusions

Significant progress has been made in the literature linking crude extracts and bioactive scaffolds from edible and medicinal plants to obesity. Up to this point, there have been many reports on the anti-obesity properties of various extracts and constituents of *Jatropha tanjorensis* and *Fraxinus micrantha*. In this work, we investigated the anti-obesity effect of ethanolic and aqueous extracts of *Jatropha tanjorensis* and *Fraxinus micrantha* leaves as we continue to focus on the anti-obesity potential of these plants. The present study claims that, compared to other extracts, the ethanolic extract of *Jatropha tanjorensis* (JTE) leaves exhibits a promising role in the control of high-fat-induced obesity. The present study provides scientific evidence and support for the traditional use of *Jatropha tanjorensis* leaf extract for the treatment of obesity.

## Figures and Tables

**Table 1:** Food intake in normal control, inducer, standard, and test samples JTAQ, JTE, FMAQ, and FME (200 and 400 mg/kg) treated groups.

Group No.	Treatment	0 Week	3 Week	6 Week
1.	Normal Control (Vehicle treated)	12.16 ± 0.820	13.33 ± 0.982	15.14 ± 0.864
2.	HFD induced only	13.52 ± 1.306	17.70 ± 1.170	23.06 ± 1.582
3.	HFD + Standard Orlistat (30 mg/kg)	12.33 ± 1.034 ^NS^	14.10 ± 1.119 **	16.07 ± 1.169 **
4.	HFD + JTAQ (200 mg/kg)	12.33 ± 0.977 ^NS^	14.71 ± 0.872 *	18.25 ± 1.249 **
5.	HFD + JTAQ (400 mg/kg)	13.37 ± 1.338 ^NS^	15.85 ± 1.611 ^NS^	18.50 ± 1.149 **
6.	HFD + JTE (200 mg/kg)	11.54 ± 1.288 ^NS^	13.47 ± 1.459 **	15.57 ± 1.688 **
7.	HFD + JTE (400 mg/kg)	12.10 ± 1.229 ^NS^	14.08 ± 1.314 **	16.28 ± 1.854 **
8.	HFD + FMAQ (200 mg/kg)	11.89 ± 1.092 ^NS^	15.35 ± 1.317 ^NS^	19.59 ± 1.166 **
9.	HFD + FMAQ (400 mg/kg)	12.45 ± 0.932 ^NS^	15.53 ± 0.911 ^NS^	18.72 ± 1.948 **
10.	HFD + FME (200 mg/kg)	13.06 ± 1.721 ^NS^	15.50 ± 1.377 ^NS^	18.52 ± 1.205 **
11.	HFD + FME (400 mg/kg)	10.99 ± 0.976 *	12.82 ± 0.641 **	14.09 ± 0.748 **

One-way ANOVA followed by the Bonferroni test, with values reported as MEAN ± SD at n = 6, * *p* < 0.050, ** *p* < 0.001, and ^NS^ *p* > 0.001 compared to group HFD treated.

**Table 2:** Variations in body weight in normal control, inducer, standard, and test samples JTAQ, JTE, FMAQ, and FME (200 and 400 mg/kg).

Group No.	Treatment	0 Week	3 Week	6 Week
I.	Normal Control (Vehicle treated)	202.58 ± 5.218	207.33 ± 5.354	214.50 ± 4.505
II.	HFD induced only	207.37 ± 5.845	215.03 ± 6.267	223.74 ± 7.090
III.	HFD + Standard Orlistat (30 mg/kg)	195.53 ± 4.398 *	199.64 ± 4.052 *	206.60 ± 3.706 **
IV.	HFD + JTAQ (200 mg/kg)	199.26 ± 4.707 ^NS^	206.63 ± 2.763 ^NS^	213.68 + 2.633 *
V.	HFD + JTAQ (400 mg/kg)	195.98 ± 4.796 *	201.33 ± 4.134 *	208.72 ± 3.824 **
VI.	HFD + JTE (200 mg/kg)	202.94 ± 2.464 ^NS^	208.60 ± 2.643 ^NS^	215.16 ± 2.639 ^NS^
VII.	HFD + JTE (400 mg/kg)	208.97 ± 3.941 ^NS^	214.45 ± 4.110 ^NS^	221.66 ± 4.718 ^NS^
VIII.	HFD + FMAQ (200 mg/kg)	194.25 ± 4.502 *	200.67 ± 4.110 *	210.82 ± 4.944 *
IX.	HFD + FMAQ (400 mg/kg)	206.46 ± 1.965 ^NS^	213.00 ± 1.965 ^NS^	220.50 ± 2.664 ^NS^
X.	HFD + FME (200 mg/kg)	206.00 ± 6.693 ^NS^	212.03 ± 5.748 ^NS^	219.74 ± 6.053 ^NS^
XI.	HFD + FME (400 mg/kg)	199.21 ± 8.699 ^NS^	205.53 ± 7.311 *	213.00 ± 6.870 *

One-way ANOVA followed by the Bonferroni test, with values reported as MEAN ± SD at n = 6, * *p* < 0.050, ** *p* < 0.001, and ^NS^ *p* > 0.001 compared to group HFD treated.

**Table 3:** Variations in blood glucose level after 0, 3, and 6 weeks of the treatment period.

Group No.	Treatment	0 Week	3 Week	6 Week
I.	Normal Control (Vehicle treated)	91.16 ± 6.911	92.83 ± 7.083	94.66 ± 5.610
II.	HFD induced only	193.33 ± 11.003	248.16 ± 12.922	286.83 ± 9.174
III.	HFD + Standard Orlistat (30 mg/kg)	188.50 ± 16.706 ^NS^	159.83 ± 8.886 **	118.00 ± 4.690 **
IV.	HFD + JTAQ (200 mg/kg)	211.00 ± 5.831 ^NS^	267.66 ± 9.026 ^NS^	200.66 ± 5.715 **
V.	HFD + JTAQ (400 mg/kg)	200.50 ± 7.232 ^NS^	233.50 ± 9.138 ^NS^	161.00 ± 13.161 **
VI.	HFD + JTE (200 mg/kg)	205.50 ± 13.576 ^NS^	228.16 ± 9.239 ^NS^	169.33 ± 11.911 **
VII.	HFD + JTE (400 mg/kg)	209.16 ± 14.634 ^NS^	189.16 ± 6.014 **	123.16 ± 4.309 **
VIII.	HFD + FMAQ (200 mg/kg)	207.08 ± 7.619 ^NS^	253.03 ± 9.826 ^NS^	211.03 ± 7.593 **
IX.	HFD + FMAQ (400 mg/kg)	192.66 ± 12.628 ^NS^	226.00 ± 14.615 *	161.33 ± 10.033 **
X.	HFD + FME (200 mg/kg)	209.83 ± 8.183 ^NS^	250.33 ± 17.294 ^NS^	193.00 ± 14.993 **
XI.	HFD + FME (400 mg/kg)	192.83 ± 16.167 ^NS^	209.83 ± 10.028 **	156.83 ± 5.776 **

One-way ANOVA followed by the Bonferroni test, with values reported as MEAN ± SD at n = 6, * *p* < 0.050, ** *p* < 0.001, and ^NS^ *p* > 0.001 compared to group HFD treated.

**Table 4:** Variations in lipid profile in normal control, inducer, standard and test samples JTAQ, JTE, FMAQ, and FME (200 and 400 mg/kg).

Group No.	Treatment	TC	TG	HDL	LDL
I	Normal Control (Vehicle treated)	100.22 ± 2.751	86.21 ± 0.967	52.81 ± 4.489	30.16 ± 6.473
II	HFD induced only	153.66 ± 2.934	191.57 ± 8.099	15.69 ± 0.667	99.65 ± 3.879
III	HFD + Standard Orlistat (30 mg/kg)	93.14 ± 2.631 **	90.35 ± 1.723 **	46.66 ± 7.242 **	28.41 ± 8.784 **
IV	HFD + JTAQ (200 mg/kg)	130.74 ± 2.673 **	111.22 ± 12.938 **	37.87 ± 3.529 **	70.61 ± 3.597 **
V	HFD + JTAQ (400 mg/kg)	112.92 ± 2.000 **	102.14 ± 2.177 **	40.90 ± 7.519 **	51.58 ± 7.774 **
VI	HFD + JTE (200 mg/kg)	119.48 ± 3.752 **	94.42 ± 2.529 **	35.75 ± 5.100 **	64.84 ± 7.082 **
VII	HFD + JTE (400 mg/kg)	107.14 ± 1.718 **	100.94 ± 2.298 **	42.42 ± 6.867 **	44.53 ± 6.396 **
VIII	HFD + FMAQ (200 mg/kg)	111.74 ± 1.706 **	137.54 ± 17.086 **	23.03 ± 2.969 ^NS^	61.20 ± 4.256 **
IX	HFD + FMAQ (400 mg/kg)	135.81 ± 0.947 **	120.35 ± 9.360 **	26.66 ± 4.833 ^NS^	85.07 ± 6.331 *
X	HFD + FME (200 mg/kg)	140.25 ± 2.105 **	122.49 ± 3.921 **	28.18 ± 7.862 *	87.57 ± 7.555 ^NS^
XI	HFD + FME (400 mg/kg)	111.74 ± 1.706 **	115.36 ± 2.530 **	30.60 ± 8.246 *	58.06 ± 7.510 **

One-way ANOVA followed by the Bonferroni test, with values reported as MEAN ± SD at n = 6, * *p* < 0.050, ** *p* < 0.001, and ^NS^ *p* > 0.001 compared to group HFD treated.

**Table 5:** Variations in oxidative markers (SOD, LPO and GSH).

Group No.	Treatment	SOD (Unit/mg Tissue)	LPO (nmol MDA/mg Tissue)	GSH (nmol/mg Tissue)
I	Normal Control (Vehicle treated)	78.85 ± 10.362	12.06 ± 0.703	6.67 ± 0.475
II	HFD induced only	13.46 ± 5.990	39.74 ± 0.825	0.85 ± 0.024
III	HFD + Standard Orlistat (30 mg/kg)	81.93 ± 10.578 **	17.35 ± 0.241 **	6.61 ± 0.245 **
IV	HFD + JTAQ (200 mg/kg)	66.29 ± 4.608 **	20.86 ± 0.948 **	4.93 ± 0.107 **
V	HFD + JTAQ (400 mg/kg)	77.95 ± 8.697 **	18.25 ± 0.675 **	4.32 ± 0.227 **
VI	HFD + JTE (200 mg/kg)	84.44 ± 10.905 **	18.70 ± 0.452 **	4.92 ± 0.010 **
VII	HFD + JTE (400 mg/kg)	106.76 ± 4.690 **	14.83 ± 0.475 **	5.02 ± 0.045 **
VIII	HFD + FMAQ (200 mg/kg)	48.05 ± 9.998 **	23.22 ± 1.045 **	3.05 ± 0.052 **
IX	HFD + FMAQ (400 mg/kg)	51.44 ± 2.728 **	22.71 ± 0.747 **	4.05 ± 0.627 **
X	HFD + FME (200 mg/kg)	56.22 ± 7.715 **	24.59 ± 0.492 **	3.93 ± 0.979 **
XI	HFD + FME (400 mg/kg)	60.41 ± 8.517 **	21.88 ± 0.825 **	4.01 ± 0.599 **

One-way ANOVA followed by the Bonferroni test, with values reported as MEAN ± SD at n = 6, ** *p* < 0.001, compared to group HFD treated.

## Data Availability

Not applicable.
